# Superovulation with an anti-inhibin monoclonal antibody improves the reproductive performance of rat strains by increasing the pregnancy rate and the litter size

**DOI:** 10.1038/s41598-024-58611-9

**Published:** 2024-04-26

**Authors:** Keiji Mochida, Kohtaro Morita, Yoshio Sasaoka, Kento Morita, Hitoshi Endo, Ayumi Hasegawa, Masahide Asano, Atsuo Ogura

**Affiliations:** 1 RIKEN BioResource Research Center, Tsukuba, Ibaraki 305-0074 Japan; 2 Institute of Laboratory Animals, Graduate School of Medicine, Kyoto University, Yoshida-Konoe-cho, Kyoto, 606-8501 Japan; 3 Center for Molecular Prevention and Environmental Medicine, Tokai University School of Medicine, Isehara, Kanagawa 259-1193 Japan; 4 Graduate School of Life and Environmental Science, University of Tsukuba, Tsukuba, Ibaraki 305-8577 Japan; 5 RIKEN Cluster for Pioneering Research, Wako, Saitama 351-0198 Japan

**Keywords:** Cell biology, Developmental biology

## Abstract

Rats are multiparous rodents that have been used extensively in research; however, the low reproductive performance of some rat strains hampers the broader use of rats as a biomedical model. In this study, the possibility of increasing the litter size after natural mating in rats through superovulation using an anti-inhibin monoclonal antibody (AIMA) was examined. In outbred Wistar rats, AIMA increased the number of ovulated oocytes by 1.3-fold. AIMA did not affect fertilization and subsequent embryonic development, resulting in a 1.4-fold increase in litter size and a high pregnancy rate (86%). In contrast, conventional superovulation by eCG/hCG administration decreased the pregnancy rate to 6–40% and did not increase the litter size. In inbred Brown Norway rats, AIMA increased the litter size by 1.2-fold, and the pregnancy rate increased more than twice (86% versus 38% in controls). AIMA also increased the litter size by 1.5-fold in inbred Tokai High Avoiders and Fischer 344 rats. AIMA increased the efficiency of offspring production by 1.5-, 2.7-, 1.4-, and 1.4-fold, respectively, in the four rat strains. Thus, AIMA may consistently improve the reproductive performance through natural mating in rats, which could promote the use of AIMA in biomedical research.

## Introduction

One strategy for improving reproductive efficiency in multiparous animals is to increase the litter size by inducing superovulation in females. The number of follicles that develop during the estrous cycle is largely determined by the balance between follicle-stimulating hormone (FSH) secreted from the pituitary gland and inhibin secreted from granulosa cells, which inhibit FSH release^1^. In small laboratory rodents, including mice, rats, and hamsters, the long-established superovulation regimen using equine chorionic gonadotrophin and human chorionic gonadotrophin (eCG/hCG) is widely used in reproductive and developmental engineering experiments, such as in vitro fertilization (IVF), intracytoplasmic sperm injection (ICSI), and nuclear transfer, because it readily increases the number of ovulated oocytes^2–8^. However, many studies have indicated that this superovulation regimen does not always increase the size of the litter after natural mating and can compromise pregnancy rates^9–18^. Consistent with these findings, the adverse effects of eCG/hCG administration on litter size in mice caused by frequent failure of fertilization in vivo and/or poor embryonic development have also been confirmed^19^. In addition to eCG/hCG treatment, anti-inhibin serum (AIS) obtained from inhibin-administered goats has been shown to have a superovulation effect in a wide range of animal species, such as hamsters^20^, cows^21^, mares^22^, guinea pigs^23^, mice^24^, goats^25^, and rats^26^. In particular, AIS-based superovulation protocols have been well optimized in mice, including conventional (classical) inbred and wild-derived strains^27–29^. However, even with different doses of AIS, the litter size in mice after natural mating does not improve^19^. The only effective way to increase litter size has been to use the recently developed anti-inhibin monoclonal antibody (AIMA). AIMA targets the same inhibin as AIS. AIMA is superior to eCG/hCG in increasing the number of ovulated oocytes and positively affecting the number of live births, with approximately 1.4 times more surviving pups than in controls. Furthermore, the AIMA-dependent superovulation protocol increased the number of knockout pups per litter after genome editing through in vivo electroporation—improved genome editing through oviductal nucleic acid delivery (*i*-GONAD)^19^.

Thus, a reliable protocol to increase the number of pups following natural mating has been successfully devised for mice; however, no such method is available for rats. Laboratory rats provide important experimental models for pharmacology, physiology, behavioral science, and psychology because of the availability of genetically defined and gene-modified strains. In addition, their larger body size makes operations and collection of more material easier than for mice. In addition, eCG/hCG administration has been widely used as a standard superovulation regimen in rats^2,3^. Recently, we proposed a new superovulation protocol for rats. It involves prior administration of a luteinizing hormone-releasing hormone (LHRH) agonist ([des-Gly10, D-Ala6]-LH-RH diethylamide acetate salt hydrate) followed by AIS, and eCG treatment synergistically increases the number of ovulated oocytes^30^. In particular, additional AIS treatment was found to increase the number of ovulations by 1.9-fold (from 22.3 to 42) in Brown Norway (BN) rats^30^. This new superovulation protocol can be applied to improve the efficiency of in vitro fertilization and generation of knock-in rats using the CRISPR/Cas9 system^30^. However, an increase in rat litter size after natural mating using the existing superovulation formula has not been reported.

These experimental findings using mice and rats lent us the impetus to examine whether AIMA can improve the efficiency of offspring production in rats. Improving the reproductive performance of rats by increasing their litter size would be a significant advantage. In this study, superovulation experiments were conducted using AIMA to examine whether it can increase the litter size of rats from different strains commonly used in biomedical research.

## Results

### Superovulation efficiency and fertilization in vivo following AIMA treatment in Wistar rats

**Figure 1:**
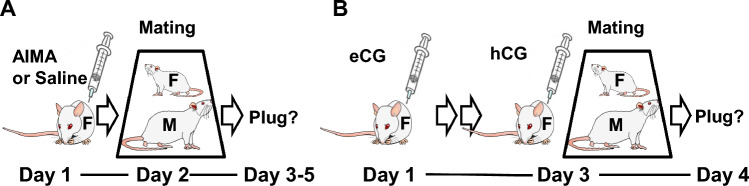
Schematic representation of superovulation treatments and mating tests. (**A**) AIMA or saline treatment. (**B**) Conventional eCG/hCG treatment. The treated females were paired with male rats on Day 2 (**A**) or Day 3 (**B**). F, female; M, male.

First, superovulation efficiency with AIMA and eCG/hCG treatments was examined in Wistar rats and compared with that of a saline-injected (control) group (Fig. 1). The ovulation rates in all the groups of females with plugs were 100% (Table 1). The numbers of ovulated and fertilized oocytes were significantly higher in the AIMA- and eCG/hCG-injected groups than in the control group (Table 1). The lower eCG/hCG dose (150/75 IU/kg) produced the highest superovulation efficiency (62.6 oocytes per female) and yielded the largest number of fertilized oocytes (55.4 fertilized oocytes per female). In contrast, a higher eCG/hCG dose (300/300 IU/kg) decreased the fertilization rate to 56%, and the number of fertilized oocytes per female did not increase from the control level (Table 1). Treatment with AIMA significantly increased the number of fertilized oocytes with the highest fertilization rate (98%; Table 1).

**Table 1:** Results of superovulation in Wistar rats after different superovulation treatments and mating.

Treatment (dose)	No. (%) of females		Mean number (%) of oocytes (± SEM)			
	With plugs	Ovulated	Total	Fertilized	Unfertilized	Abnormal
Control (Saline 0.2 mL)	7	7 (100)	14.1 ± 1.0^a^	13.0 ± 1.0^a^ (92)	1.0 ± 0.4^a^ (7)	0.1 ± 0.1 (1)
eCG/hCG (150/75 IU/kg)	8	8 (100)	62.6 ± 7.2^b^	55.4 ± 9.4^b^ (88)	7.3 ± 2.9 (12)	0 ± 0 (0)
eCG/hCG (300/300 IU/kg)	8	8 (100)	35.1 ± 6.4^b^	19.6 ± 4.6 (56)	15.1 ± 3.4^b^ (43)	0.4 ± 0.4 (1)
AIMA (0.2 mL)	8	8 (100)	18.4 ± 0.9^b^	18.1 ± 1.0^b^ (98)	0 ± 0 (0)	0.3 ± 0.2 (2)

Numbers with different superscript within the same column are significantly different (^a,b^*P* < 0.01, vs. control, One-way ANOVA with post hoc *t* test).

### Pregnancy, embryo development in vivo, and the birth of pups following AIMA treatment in Wistar rats

**Figure 2:**
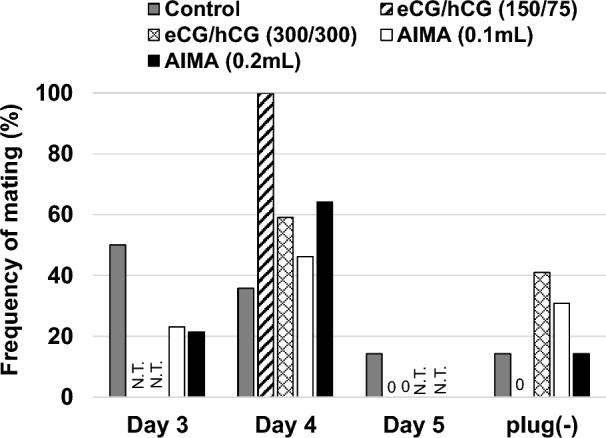
Distribution of appearance of the days of vaginal plug during pairing with experienced male rats. Before mating, the females were treated with 0.1 mL (white bar, N = 13) or 0.2 mL (black bar, N = 14) of AIMA, 150/75 IU/kg (diagonal line bar, N = 10) or 300/300 IU/kg (lattice line bar, N = 17) of eCG/hCG, or saline (gray bar, N = 16) as control.* N.T*. not tested.

**Figure 3:**
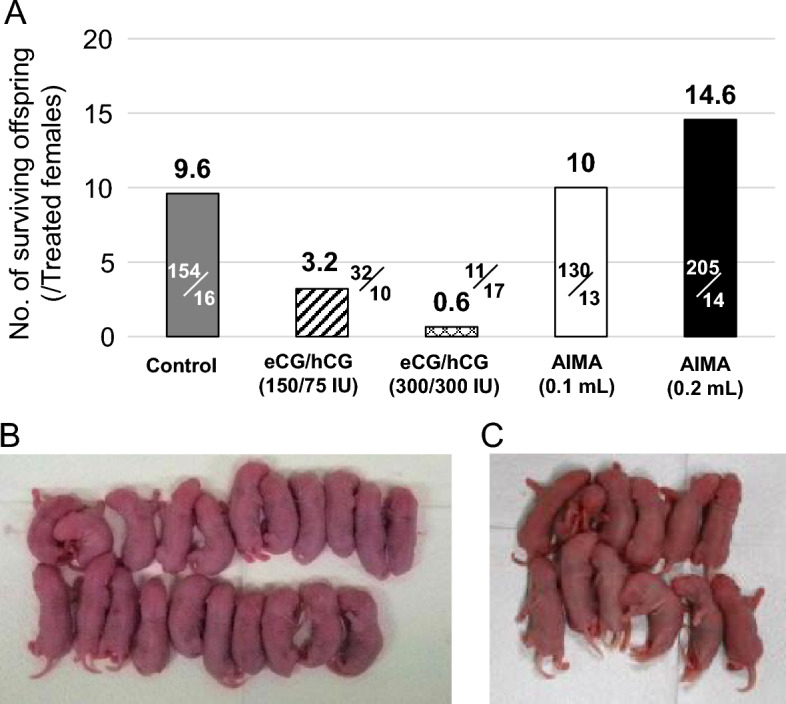
Births and offspring development following superovulation treatments and mating with males in Wistar rats. (**A**) Total yields of surviving offspring per treated female following different superovulation treatments. (**B**) The highest litter size in the AIMA treatment group. (**C**) Example of an average litter size in the control group.

Next, the effect of AIMA treatment on pregnancy rates after mating was examined. Females superovulated with conventional eCG/hCG treatment frequently do not become pregnant, even after successful mating^10,11^. After standard treatment (0.2 mL) with AIMA, followed by mating with males for 3 days, 86% (12/14) of the treated females exhibited a vaginal plug during this period, and all (12/12) became pregnant (Fig. 2, Table 2). In addition, a half volume (0.1 mL) of AIMA was tested, and the mating rate decreased to 69% (9/13) (Fig. 2, Table 2). At both doses, the peak of mating was observed on Day 4, 1 day later than the peak on Day 3 in the saline group (Fig. 2). The size of the litter was largest in the standard AIMA group (17.1 pups), which was significantly higher than that in the control group (11.8 pups; Table 2). In contrast, only 10% (1/10)–40% (4/10) of the eCG/hCG-treated and mated females became pregnant, corresponding to only 6% (1/17) of the treated females in the higher-dose eCG/hCG (300/300 IU/kg) group. In the lower-dose eCG/hCG (150/75 IU/kg) group, the survival rate after implantation to term was lower than that in the control group despite having the highest number of implantation sites (20.8). No eCG/hCG treatment group showed an increase in the average number of pups per female compared with the control group (Table 2). When calculated based on the number of treated females, the number of pups per litter was 14.6 in the standard AIMA group—that is, 1.5 times higher than the 9.6 in the control group (Fig. 3A).

**Table 2:** Production of offspring after different superovulation treatments in Wistar rats.

Treatment (dose)	No. (%) of females			Implantation sites per female (total no.)	Survival rate after imp. to term (%)	Litter size		Survival rate (%) (E/D)
	Treated A	With plug B (B/A)	Pregnant C (C/B)			Living D	Surviving E	
Control (saline 0.1–0.2 mL)	16	14 (88)	13 (93)^a^	12.2 ± 0.9 (158)^A^	154/158 (97)^a^	11.8 ± 0.9^a^	11.8 ± 0.9^a^	100
eCG/hCG (150/75 IU/kg)	10	10 (100)^A^	4 (40)^b,A^	20.8 ± 7.1 (83)^B^	32/83 (39)^b^	8.0 ± 3.5^a,A^	8.0 ± 3.5^a,A^	100
eCG/hCG (300/300 IU/kg)	17	10 (59)^B^	1 (10)^b^	13 ± 0 (13)	11/13 (85)	11 ± 0	11 ± 0	100
AIMA (0.1 mL)	13	9 (69)	9 (100)^a,B^	15.1 ± 0.9 (136)	130/136 (96)^a^	14.4 ± 0.8^B^	14.3 ± 0.8^B^	99
AIMA (0.2 mL)	14	12 (86)	12 (100)^a^	18.4 ± 0.7 (221)^A^	205/221 (93)^a^	17.1 ± 0.7^b^	17.1 ± 0.7^b^	100

Fisher’s exact probability test analyzed the percentages of females with plug, pregnant, and survival rates. One-way ANOVA with Tukey’s post hoc multiple comparison test analyzed the number of implantation sites per female and litter size (mean ± SEM). Numbers with different superscripts within the same column are significantly different (^a,b^*P* < 0.01, ^A,B^*P* < 0.05).

The serum estradiol (E2) and progesterone (P4) levels on Day 5 of pregnancy (the day a vaginal plug was recovered was designated Day 1) were examined to determine the hormonal dynamics of early pregnancy. Although there were no statistically significant differences in E2 and P4 levels between the groups, in the AIMA and eCG groups, P4 levels increased compared with the control level (Supplementary Fig. S1).

Birth weights were significantly lower in the AIMA and eCG/hCG groups than in the control group (Table 3). However, the pups in the AIMA group recovered their body weights to levels comparable to those of the control group at 1.5 and 3 weeks of age (Table 3). The body weights of the eCG/hCG group remained significantly lower than those of the control group until at least 3 weeks of age (Table 3). The weaning rate of the surviving pups was 84% (154/184) in the AIMA group, which was comparable to that in the control group (83%, 85/103; Table 3, Fig. 3B,C). A mating test was conducted with four pairs of pups born in the AIMA and control groups, and all pairs produced litters and were nursed until weaning. Three of the pairs were reused for the second mating cycle and pregnancy. The females gave birth to normal pups and nursed them until weaning. Thus, the pups produced following the AIMA treatment had normal reproductive performance.

**Table 3:** Offspring development in Wistar rats after different superovulation treatments.

Treatment	No. (%) of offspring		Body weight (0 W)			Body weight (1.5 W)			Body weight (3 W)		
	Fostered A	Weaned B (B/A)	Female	Male	Total	Female	Male	Total	Female	Male	Total
Control (0.1–0.2 mL)	103	85 (83)^a^	6.58 ± 0.05^a^	6.79 ± 0.07^a^	6.70 ± 0.05^a^	22.1 ± 0.4	22.5 ± 0.3^a^	22.4 ± 0.3^a^	51.1 ± 0.9^A^	52.9 ± 0.9^a^	52.3 ± 0.7 ^a^
eCG/hCG (150/75 IU/kg)	32	32 (100)^A,b^	6.46 ± 0.20^a^	5.79 ± 0.30^b^	6.19 ± 0.18^b^	20.7 ± 0.4^A^	19.7 ± 0.7^b^	20.3 ± 0.4^b^	47.6 ± 1.0^a, B^	46.3 ± 1.2^b^	47.1 ± 0.8 ^b^
AIMA (0.2 mL)	112	94 (84)^B^	5.90 ± 0.07^b^	6.15 ± 0.08^b^	6.03 ± 0.06^b^	22.0 ± 0.4^B^	23.3 ± 0.4^a^	22.7 ± 0.3^a^	51.9 ± 0.9^b^	54.6 ± 0.9^a^	53.5 ± 0.6 ^a^

The body weights were measured using 47, 32 and 74 offspring in control, eCG/hCG and AIMA groups, respectively. Fisher’s exact probability test analyzed weaning rates. One-way ANOVA with Tukey’s post hoc multiple comparison test analyzed the body weight of the offspring (mean ± SEM). Numbers with different superscripts within the same column are significantly different (^a,b^*P* < 0.01, ^A,B^*P* < 0.05).

### Effects of AIMA treatment on litter size and number of surviving pups in three inbred strains

**Figure 4:**
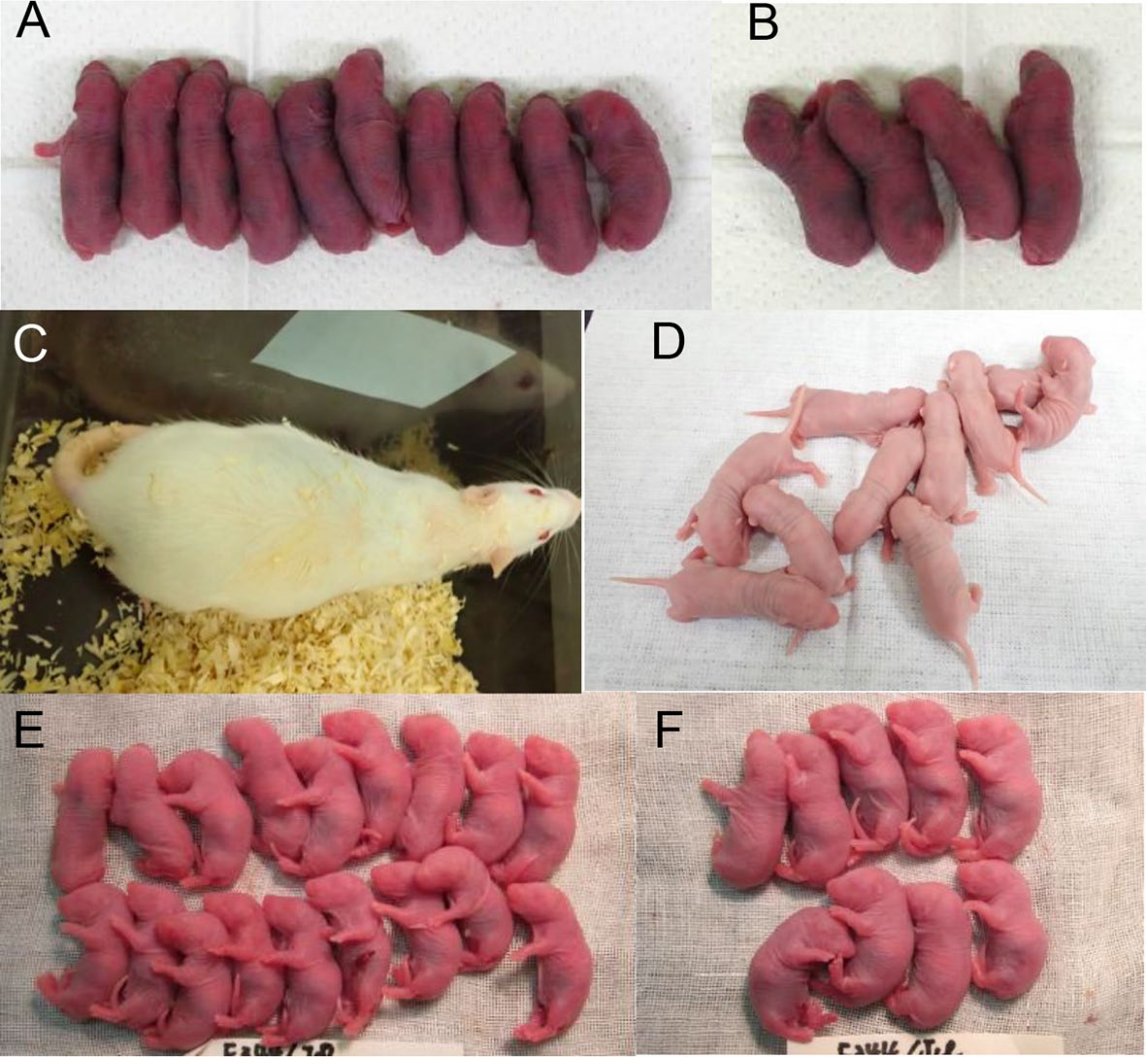
Surviving pups after cesarean section from pregnant females after superovulation treatment with AIMA and mating with males. Examples of the highest (**A**) and lowest (**B**) litter sizes in the AIMA treatment group of the BN strain. THA pregnant female rat 2 days before delivery (**C**), and offspring obtained by AIMA treatment (**D**). Examples of the highest (**E**) and lowest (**F**) litter sizes in the AIMA treatment group of the F344 strain.

The effects of AIMA treatment on inbred strains were examined next. In the inbred BN strain, only 38% (3/8) of the females in the control group became pregnant, while 86% (6/7) of the AIMA-treated females became pregnant (Table 4). The average number of pups per litter was 7.2 in the AIMA-treated group, which was slightly larger than that of the control group (6.0) but not significantly different (Table 4, Fig. 4A,B). However, when the numbers of pups were compared based on the treated females, the AIMA treatment produced 2.7 times more pups (6.1 pups) compared with 2.3 pups in the control group (see below).

**Table 4:** Offspring production after AIMA treatment in BN rats.

Treatment	No. (%) of females			Implantation sites per female (total no.)	Survival rate after imp. to term (%)	Litter size		Survival rate (%) (E/D)
	Treated A	With plug B (B/A)	Pregnant C (C/B)			Living D	Surviving E	
Control (saline 0.2 mL)	8	7 (80)	3 (43)	7.0 ± 0 (21)	18/21 (86)	6.0 ± 0.6	6.0 ± 0.6	100
AIMA (0.2 mL)	7	7 (100)	6 (86)	9.2 ± 1.1 (55)	43/55 (78)	7.2 ± 1.0	7.2 ± 1.0	100

There were no significant differences between the two groups.

Next, the effect of AIMA treatment on the Tokai High Avoiders (THA) strain was examined, resulting in poor reproductive performance because of a low pregnancy rate. Although the AIMA treatment did not increase successful mating (with a vaginal plug) and pregnancy rates, it increased the number of pups per litter from 8.8 to 13.2, corresponding to a 1.5-fold increase (Table 5, Fig. 4C,D).

**Table 5:** Offspring production after AIMA treatment in THA rats.

Treatment	No. (%) of females			Implantation sites per female (total no.)	Survival rate after imp. to term (%)	Litter size		Survival rate (%) (E/D)
	Treated A	With plug B (B/A)	Pregnant C (C/B)			Living D	Surviving E	
Control^#^	22	14 (64)	12 (86)	N.T	N.T	N.T	8.8 ± 0.7^A^	N.T
AIMA (0.2 mL)	10	6 (60)	5 (83)	N.T	N.T	N.T	13.2 ± 1.5^B^	N.T

Fisher’s exact probability test analyzed the percentages of females with plug and pregnant rates. Student’s *t* test analyzed the number of litter sizes per female (mean ± SEM) to compare the control results (^A,B^*P* < 0.05). *N.T.* not tested. ^#^All data are the results of natural delivery, and control data are from female rats without treatment in the breeding colony of Tokai University.

Finally, the effect of AIMA treatment in a different inbred strain, F344, was examined. The pregnancy and embryo survival rates after implantation were high (90% and 98%, respectively) after the AIMA treatment. In particular, the AIMA treatment increased the number of pups per litter from 8.9 to 13.4, a 1.5-fold increase (Supplementary Table S1, Fig. 4E,F).

### Treatment with AIMA improves the overall efficiency of offspring production in four strains of rats

**Figure 5:**
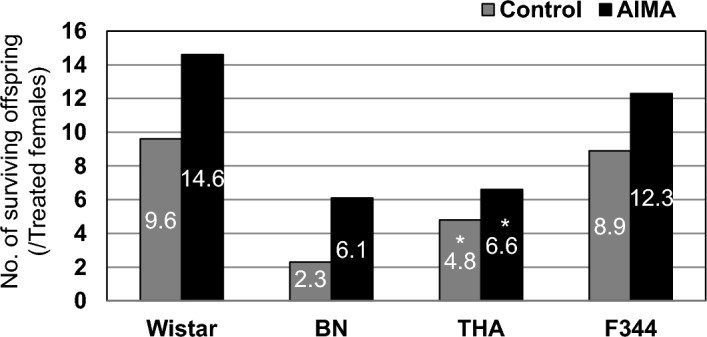
Total yields of surviving offspring per treated female following AIMA treatment in each rat strain. *All data from THA rats are the results of natural delivery in female rats at the breeding colony of Tokai University.

From a practical perspective, it is important to determine the overall efficiency of offspring production based on the number of females used for breeding. This can be calculated by considering the rates of mating (i.e., the presence of a vaginal plug) and successful pregnancy. The resultant overall efficiency (i.e., the expected number of surviving pups per female) for each rat strain is shown in Fig. 5. The AIMA treatment yielded 1.5-, 2.7-, 1.4-, and 1.4-fold increases in the number of surviving pups in the Wistar, BN, THA, and F344 strains, respectively. These results contrast sharply with those of the eCG/hCG treatment, which greatly reduced the number of surviving offspring to less than 3.2 (Wistar rats, Fig. 3A). These data clearly show the high efficacy of AIMA in producing offspring by natural mating in different rat strains.

## Discussion

The laboratory rat has a history of more than 100 years as a laboratory animal and has been used in a wide range of fields, including pharmacology, physiology, behavioral science, and psychology. Therefore, rat experiments can be performed under rigorously controlled genetic and environmental conditions based on abundant pharmacological and toxicological data. Furthermore, their large body size makes surgical procedures, transplantation experiments, and material collection easier than in mice. However, reproductive techniques for rats, especially those for inbred strains, have not been developed as intensively as for mice. In this study, the number of litters produced by natural mating of rats, including inbred strains, was increased using AIMA. In Wistar (outbred), BN (inbred), THA (inbred), and F344 (inbred) strains, AIMA-induced superovulation increased the litter size by 1.5-, 2.7-, 1.4-, and 1.4-fold, respectively (Fig. 5). Because these outbred or inbred strains have given rise to many genetically modified (knockout and knock-in) or spontaneous mutant strains^31,32^, AIMA technology is also beneficial for breeding these mutant strains, especially those with low fertility. Moreover, although the experiments reported here were conducted at three different facilities, the AIMA treatment consistently increased litter size, irrespective of the strain, breeding conditions, and operators.

It is important to understand which improvements in the steps from ovulation to birth contributed to the increase in the number of newborns after the AIMA treatment. According to experiments using Wistar rats, the number of ovulated oocytes increased by 1.3-fold (18.4 versus 14.1). All the following parameters (number of fertilized oocytes, implantation sites, and living pups) increased 1.4–1.5 times. These data indicate that the superovulation efficiency of AIMA (0.2 mL per female) directly determines the litter size in Wistar rats without any adverse effects on fertilization in vivo, pregnancy, or embryonic development. In contrast, eCG/hCG treatment decreased many reproduction-related parameters, especially the pregnancy rate (only 10–40% of females with a vaginal plug became pregnant) and survival rate (after implantation to term). When the eCG/hCG dose was reduced to 150/75 IU/kg, the numbers of ovulated oocytes, fertilized eggs, and implantation sites increased; however, the number of fetuses did not. This is consistent with the results of our mouse experiments that showed a 40% pregnancy rate and a decrease in litter size after eCG/hCG treatment. This indicates that, although eCG and/or hCG injections may induce the ovulation of more oocytes at the optimal dose, they can still lead to unexpected alterations in systemic conditions in females unsuitable for natural conception. Indeed, eCG/hCG is known to decrease implantation and fetal growth rates^9–12,14–16^ and cause a reduction in the mean size of trophoblasts^12^, delayed fetal development, and failure in the formation of pinopodes at the implantation sites^17^. These adverse effects may be, at least in part, related to the alteration of uterine receptivity (implantation window) by eCG/hCG treatment. In contrast, the AIMA used in this study is not an exogenous superovulation reagent but prevents the blockage of FSH secretion by inhibin, which naturally increases the endogenous level of FSH and, thus, does not compromise embryo/fetal development. Another notable effect of AIMA was found in inbred BN rats, in which AIMA improved the pregnancy rate from 43% (control) to 86%, leading to an improvement in the overall efficiency of offspring production in AIMA-treated BN rats. It would be interesting to determine whether AIMA can rescue other rat strains with low pregnancy rates.

While AIMA proved effective in increasing pup production through natural mating in rats and mice, a notable technical distinction exists between the two species. In mice, we synchronized their estrous cycle by administering progesterone to randomly selected females, followed by an AIMA injection 3 days later. The progesterone regimen successfully induced metestrus in 93% of treated females^29^. Conversely, in rats, progesterone injection was unnecessary, and we could directly administer AIMA to females during metestrus or diestrus, identified through vaginal smears. As anticipated, these AIMA-treated rats could mate successfully with males on the third or fourth-day post-injection. Thus, similar to mice, successful mating in rats could be achieved by housing them consecutively with males immediately after AIMA administration, without hCG treatment. Regarding estrous cycle synchronization in rats, a straightforward method involves producing pseudopregnant female rats using an LHRH agonist^33^. Consequently, AIMA can also effectively synchronize the estrous cycle in rats by preceding the injection of the LHRH agonist.

An important application of AIMA in rats is the production of genome-edited rats using *i*-GONAD, an in vivo transfection technology^34^. The *i*-GONAD method often causes a reduction in the number of pups owing to electric shock and unwanted genetic modifications^19^. Superovulation treatment with eCG/hCG does not overcome this problem, as estimated from the experiment (Fig. 3A) and other results^16^. For this reason, the current standard *i*-GONAD protocol for rats does not include superovulation treatment^16,35^. It was reported previously that AIMA administration significantly increased the number of offspring from 4.8 to 7.3 after *i*-GONAD without affecting the genome-editing efficiency in mice^19^. Therefore, one can expect that AIMA treatment in rats will also improve *i*-GONAD-mediated genome editing by increasing the number of offspring. This strategy may be particularly effective for inbred strains, such as BN, with a low reproductive potential.

The litter size was increased in all four rat strains tested by AIMA treatment, followed by natural mating. One issue that should be examined in future studies is the long-term effects of AIMA. Catch-up growth was observed in AIMA-derived pups up to 1.5 weeks of age (Table 3). This can have negative effects on metabolic health and neurological outcomes. Whether this has a transgenerational effect should also be investigated carefully in future studies.

In conclusion, because IVF and embryo transfer efficiency do not reach practical levels in many rat strains^30^, the AIMA treatment in this study combined with natural mating may maximize the reproductive performance of rats and promote their efficient use as research models in biomedical research. Furthermore, this treatment can lead to a reduction in cost and effort and fulfill the 3R principles of animal experimentation: reduction, replacement, and refinement.

## Materials and methods

### Animals

In this study, Wistar (Crlj:WI, Jackson Laboratory Japan, Kanagawa, Japan), BN (BN/SsNslc, Japan SLC Inc., Shizuoka, Japan), THA (maintained at Tokai University), and Fischer 344 (F344/Jcl, CLEA Japan Inc., Tokyo, Japan)^36–38^ strains of rats were used. Wistar rats are maintained by outbred breeding, whereas BN, THA, and F344 rats are maintained by inbred breeding. In this study, Wistar rats were used to determine the effects of different superovulation protocols on all relevant parameters, including the number of ovulated oocytes, mating rate, in vivo fertilization rate, pregnancy rate, implantation rate, and litter size. BN, THA, and F344 rats were used to examine the effectiveness of the protocol devised in Wistar rats. Female rats were 8–16 weeks old at the time of the experiments, and male rats were > 14 weeks old. All rats were maintained under specific pathogen-free conditions, provided with water and commercial laboratory mouse chow ad libitum, and housed under a controlled lighting condition (daily light period, 7:00 a.m. to 7:00 p.m.) at a temperature of 23 ± 2 °C and humidity of 50 ± 10%. The Animal Experimentation Committee of Kyoto University approved the experiments using the Wistar and BN strains. The Animal Experimentation Committee of Tokai University approved the experiments using the THA strain. The experiments using the F344 strain were approved by the Animal Experimentation Committee of CLEA Japan, Inc. All animal experiments and methods were performed in accordance with the relevant guidelines and regulations. All experiments followed the committees’ guiding principles in compliance with the ARRIVE guidelines.

### Superovulation treatments and mating with males

Female rats at the metestrus or diestrus stage were selected using a vaginal smear test^39^. The AIMA developed by RIKEN and CLEA Japan Inc. (5 mg/mL, BioGate Co., Ltd., Gifu, Japan)^19^ and eCG (ASKA Pharmaceutical Co. Ltd., Tokyo, Japan) were then used as conventional superovulation reagents for superovulation treatments. Female Wistar rats were treated by intraperitoneal injections of AIMA (0.1 or 0.2 mL, approximately 2.5 or 5 mg/kg) in the evening (3:30–6:00 p.m.) on Day 1 and paired with sexually mature and experienced male rats (1:1) on Day 2 (Fig. 1A). Saline was administered instead of AIMA to the control group. For the AIMA and control (injected with saline) groups, female rats were monitored in the morning for the next 3 days until a vaginal plug was found. Female Wistar rats for conventional superovulation treatment were administered 150 or 300 IU/kg eCG in the evening (3:30–6:00 p.m.) on Day 1, followed by injection with 75 or 300 IU/kg hCG (Gonatropin, Sankyo Co. Ltd., Tokyo, Japan) in the evening (3:30–6:00 p.m.) on Day 3 and paired with males (Fig. 1A). These females injected with eCG/hCG were examined for the presence of a vaginal plug on the morning of Day 4 following the hCG injection.

### Confirmation of the number of fertilized oocytes

In the first experiment using Wistar rats, females were treated with AIMA, eCG/hCG (150/75 or 300/300 IU/kg), or saline (control) and examined for the fertilization rates of oocytes in vivo. The ovulated and fertilized oocytes were collected and counted after puncturing the oviducts with a 26G needle or perfusing the oviducts with a 30G round-shaped needle using EmbryoMax Advanced KSOM medium (MR-101-D; Sigma-Aldrich, St. Louis, MO, USA) on the day the vaginal plug was observed.

### Examinations of pups after birth

The numbers of full-term fetuses and live pups were examined after cesarean section or natural delivery for all four strains. Pregnant females for the cesarean section (Wistar, BN, and AIMA-treated F344 rats) were injected subcutaneously with 4 mg of progesterone on the evenings of Days 20 and 21 to prevent natural delivery. At the cesarean section (10:00 a.m. to 6:00 p.m. on Day 22), pregnant females were euthanized and examined for the presence of implantation sites and live fetuses. Among the live fetuses retrieved from the uteri, those that started normal respiration after stimulation were defined as “surviving” pups. Some surviving pups (a group ≤ 15) in Wistar rats were nursed by foster mothers of the same strain for approximately 3 weeks until weaning and tested for reproductive ability after maturity. Their weights were measured at birth and at 1.5 and 3 weeks of age. After weaning, the animals were subjected to fertility tests by mating females and males of the same litter. The BN rat experiments were performed at Kyoto University as with the Wistar rats but without postnatal observations. The experiments using THA rats were performed at Tokai University, and control data were obtained from female rats without treatment in the breeding colony of Tokai University. The experiments using F344 rats were performed at CLEA Japan, Inc. In the control data for the F344 strain, a cesarean section was performed at Days 15–17 of gestation, so the fetuses alive at that time were counted as living fetuses.

### Measurement of serum estradiol and progesterone levels

Approximately 500 μL of blood was collected from veins in the tails of female rats in the control, AIMA (0.2 mL), and eCG/hCG (150/75 IU/kg) groups on Day 5 of pregnancy (N = 4 for each group). Serum levels of estradiol and progesterone were measured using liquid chromatography–tandem mass spectrometry.

### Statistical analysis

The numbers of implantation sites, pups per litter, and surviving pups and the body weights of pups in Wistar rats were analyzed using one-way analysis of variance (ANOVA), followed by Tukey’s post-hoc multiple comparison test. The average numbers of oocytes were analyzed by ANOVA followed by post-hoc *t-*test. The surviving litter size, except for Wistar rats, was analyzed using Student’s *t*-test. Ovulation, pregnancy, implantation, surviving pups, and weaning rates were analyzed using Fisher’s exact probability test. The correlations between serum estradiol/progesterone levels and the number of implantation sites were analyzed using Pearson’s correlation coefficient, and *P* < 0.05 was considered statistically significant.

### Supplementary Information

Supplementary Information.

## Data Availability

The datasets used and/or analyzed during the current study are available from the corresponding author upon reasonable request.
