# Supplementary material for: Superovulation with an anti-inhibin monoclonal antibody improves the reproductive performance of rat strains by increasing the pregnancy rate and the litter size
Source: Sci Rep. 2024 Apr 26;14:8294. doi: 10.1038/s41598-024-58611-9 (PMC11052992; doi:10.1038/s41598-024-58611-9)
Supplement: Supplementary file 1 — Supplementary Information. [file 41598_2024_58611_MOESM1_ESM.pdf]

Supplementary Information

Superovulation with an anti-inhibin monoclonal antibody improves the reproductive performance of rats by increasing the pregnancy rate and litter size

Keiji Mochida[1,†*], Kohtaro Morita[2,†], Yoshio Sasaoka[2], Kento Morita[2], Hitoshi Endo[3], Ayumi Hasegawa[1], Masahide Asano[2*], and Atsuo Ogura[1,4,5*]

[1] *RIKEN BioResource Research Center, Tsukuba, Ibaraki 305-0074, Japan*
[2] *Institute of Laboratory Animals, Graduate School of Medicine, Kyoto University, Yoshida-Konoe-cho, Kyoto 606-8501, Japan*
[3] *Center for Molecular Prevention and Environmental Medicine, Tokai University School of Medicine, Isehara, Kanagawa 259-1193, Japan*
[4] *Graduate School of Life and Environmental Science, University of Tsukuba, Tsukuba, Ibaraki 305-8577, Japan*
[5] *RIKEN Cluster for Pioneering Research, Wako, Saitama 351-0198, Japan*
[†] These authors contributed equally: Keiji Mochida and Kohtaro Morita.
*Correspondence: K. Mochida (e-mail: keiji.mochida@riken.jp), A. Ogura (e-mail: ogura@rtc.riken.go.jp), and M. Asano (e-mail:asano.masahide.5u@kyoto-u.ac.jp).

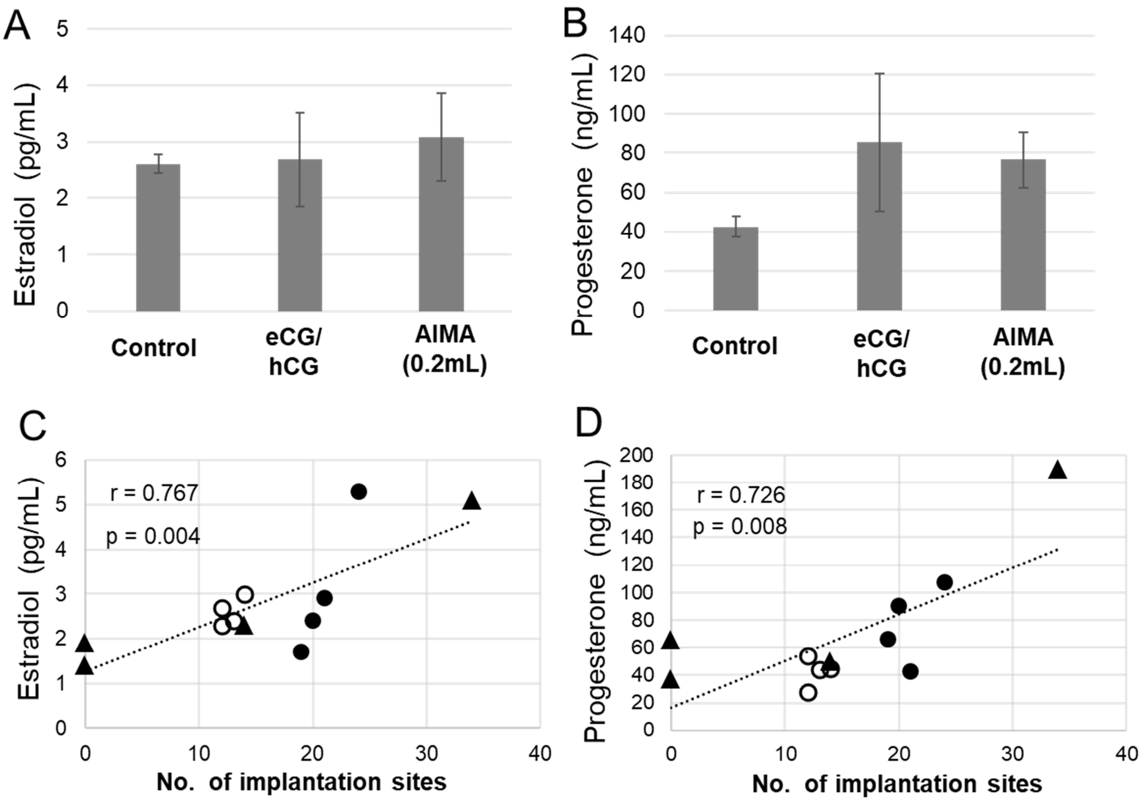

Supplementary Figure S1. Serum levels of estradiol and progesterone in Wistar rats after different superovulation treatments. Means ± SEM of estradiol (**A**) and progesterone (**B**) levels and each correlation with the number of implantation sites (**C**, **D**) from females treated with AIMA (0.2 mL, closed circles), eCG/hCG (150/75 IU/Kg, triangles), and saline as control (open circles) at Day 5 of pregnancy. There are no significant differences in estradiol or progesterone levels between the three groups.

Supplementary Table S1. Offspring production after AIMA treatment in F344 rats.

Treatment	No. (%) of females			Implantation sites per female (total no.)	Survival rate after imp. to term (%)	Litter size		Survival rate (%) (E/D)
	Treated A	With plug B (B/A)	Pregnant C (C/B)			Living D	Surviving E	
Control*	7	7 (100)	7 (100)	9.9 ± 0.8 (69)[a]	N.T.	8.9 ± 1.1[a]	N.T.	N.T.
AIMA (0.2 mL)	10	10 (100)	9 (90)	13.8 ± 1.0 (124)[b]	123/124 (99)	13.7 ± 1.0[b]	13.4 ± 1.0[b]	98

The average number of surviving litter sizes (mean ± SEM) was analyzed using Student's *t* test. [a-b] $P < 0.01$. N.T., not tested.

* The control data were confirmed by cesarean section at 15-17 days gestation without treatment at the CLEA Japan Inc. breeding colony.
